# Inhibiting the urokinase‐type plasminogen activator receptor system recovers STZ‐induced diabetic nephropathy

**DOI:** 10.1111/jcmm.14004

**Published:** 2018-11-13

**Authors:** Massimo Dal Monte, Maurizio Cammalleri, Valeria Pecci, Monica Carmosino, Giuseppe Procino, Alessandro Pini, Mario De Rosa, Vincenzo Pavone, Maria Svelto, Paola Bagnoli

**Affiliations:** ^1^ Department of Biology University of Pisa Pisa Italy; ^2^ Department of Sciences University of Basilicata Potenza Italy; ^3^ Department of Biosciences, Biotechnologies and Biopharmaceutics University of Bari Bari Italy; ^4^ Department of Experimental and Clinical Medicine University of Firenze Firenze Italy; ^5^ Department of Experimental Medicine Second University of Napoli Napoli Italy; ^6^ Department of Chemical Sciences University of Napoli Federico II Napoli Italy; ^7^ Institute of Biomembranes and Bioenergetics National Research Council Bari Italy

**Keywords:** αvβ3 integrin/Rac‐1 pathway, AQP2 expression and localization, diabetic kidney disease, ECM markers and renal fibrosis, glomerular morphology and filtration barrier, inflammation markers, standard renal parameters, uPAR pathway, UPARANT (Cenupatide), vascular permeability

## Abstract

The urokinase‐type plasminogen activator (uPA) receptor (uPAR) participates to the mechanisms causing renal damage in response to hyperglycaemia. The main function of uPAR in podocytes (as well as soluble uPAR ‐(s)uPAR‐ from circulation) is to regulate podocyte function through αvβ3 integrin/Rac‐1. We addressed the question of whether blocking the uPAR pathway with the small peptide UPARANT, which inhibits uPAR binding to the formyl peptide receptors (FPRs) can improve kidney lesions in a rat model of streptozotocin (STZ)‐induced diabetes. The concentration of systemically administered UPARANT was measured in the plasma, in kidney and liver extracts and UPARANT effects on dysregulated uPAR pathway, αvβ3 integrin/Rac‐1 activity, renal fibrosis and kidney morphology were determined. UPARANT was found to revert STZ‐induced up‐regulation of uPA levels and activity, while uPAR on podocytes and (s)uPAR were unaffected. In glomeruli, UPARANT inhibited FPR2 expression suggesting that the drug may act downstream uPAR, and recovered the increased activity of the αvβ3 integrin/Rac‐1 pathway indicating a major role of uPAR in regulating podocyte function. At the functional level, UPARANT was shown to ameliorate: (a) the standard renal parameters, (b) the vascular permeability, (c) the renal inflammation, (d) the renal fibrosis including dysregulated plasminogen‐plasmin system, extracellular matrix accumulation and glomerular fibrotic areas and (e) morphological alterations of the glomerulus including diseased filtration barrier. These results provide the first demonstration that blocking the uPAR pathway can improve diabetic kidney lesion in the STZ model, thus suggesting the uPA/uPAR system as a promising target for the development of novel uPAR‐targeting approaches.

## 1 INTRODUCTION

Diabetic nephropathy (DN) is a microvascular complication of diabetes leading to end‐stage renal disease that is difficult to handle despite strict glycaemic control and targeted therapies, thus indicating the paramount importance to develop novel treatments. DN can be reproduced in the rat model of type‐1 diabetes induced by streptozotocin (STZ)^1^ with main alterations that are established 4 weeks after diabetes onset.^2^ They include glomerular hypertrophy and altered filtration barrier that is associated to increased albumin and creatinine excretion.^3^ In the medulla, increased glomerular filtration results in up‐regulated levels of the main transporter proteins involved in urine concentration among which aquaporin 2 (AQP2) concurs to prevent excessive water loss.^4^, ^5^ At the glomerular level, additional alterations include an increased mesangial area and renal fibrosis induced by excessive accumulation of extracellular matrix (ECM) presumably because of inflammatory processes activated by transcriptional regulators of inflammation‐related genes.^6^, ^7^

Major players in the diabetes‐associated renal fibrosis include members of the plasminogen (Plg)‐plasmin system that consists of the circulating zymogen Plg and its activators including the urokinase‐type plasminogen activator (uPA), a secreted protease that, through the binding to its receptor (uPAR), converts Plg into plasmin that promotes ECM degradation either directly or indirectly through the activation of metalloproteinases (MMPs).^8^ uPA is a secreted protease, while uPAR is expressed by glomerular cells, resident fibroblasts and cells of the collecting ducts.^9^ In rodent models of DN, uPA and uPAR are both up‐regulated in glomerular cells including podocytes suggesting that dysfunction of the uPA/uPAR system may be associated with kidney disease.^10^ In contrast, uPA or uPAR deletion results in protective effects against kidney injuries.^9^

uPAR also exists in a soluble form ((s)uPAR) that is generated by the proteolytic cleavage of the membrane anchored uPAR.^10^ In particular, (s)uPAR has been recently demonstrated as a key molecule in the diseased kidney^10^, ^11^ and high (s)uPAR levels in the circulation play a massive role in diabetic kidney disease by regulating podocyte function.^12^ Indeed, vast data are available on major role of uPAR in podocytes (as well as (s)uPAR from circulation) to regulate the activity of αvβ3 integrin that in turns stimulates small GTPase Rac‐1 proteins, potent regulators of podocyte foot process motility and effacement.^13^, ^14^ Altered activity of αvβ3 integrin/Rac‐1 pathway has been linked to podocyte dysfunction leading to proteinuria^12^ and main efforts have been made to clarify mechanisms for uPAR signalling in regulating podocyte adhesion and migration.^10^

At the intracellular level, the interaction between uPA and uPAR is mediated by several membrane proteins including the formyl peptide receptors (FPRs) that are G protein‐coupled receptors involved in different pathophysiological processes,^8^ although little is known about their possible involvement in diabetic kidney disease. The transmembrane partnership between uPA, uPAR and FPRs has been reported as an attractive target for the treatment of DN, the role of drugs potentially interacting with the uPAR/FPR pathway remains to be established. The uPAR‐derived tetrapeptide Ac‐L‐Arg‐Aib‐L‐Arg‐L‐Cα(Me)PheNH_2_ (UPARANT, recently designated as Cenupatide in the International Nonproprietary Names nomenclature) has been designed to compete with N‐formyl‐Met‐Leu‐Phe peptide for binding to FPRs.^15^ It is endowed with a significant anti‐inflammatory and antiangiogenic activity both in vitro and in vivo^15^, ^16^, ^17^, ^18^, ^19^, ^20^ and has been shown to protect the retina from pathologic changes induced by diabetic retinopathy (DR) in animal models.^21^, ^22^ In this scenario, the possibility that dysregulated uPAR pathway participates to the pathogenic mechanisms of DN may add further value to the possible development of UPARANT as valuable therapy against diabetes complications.

Here, we evaluated the curative effects of subcutaneously administered UPARANT on diabetic kidney disease using rats with STZ‐induced diabetes. In the present study, UPARANT concentration in the plasma, kidney and liver was determined. Protein levels of uPA, uPAR and FPRs were measured in kidney extracts, while transcripts of FPRs were determined in isolated glomeruli, before and after UPARANT treatment. UPARANT effects on (s)uPAR were also evaluated. In addition, protein levels and activity of uPA, Plg, MMP‐2/MMP‐9 were measured in kidney extracts and in the plasma. Finally, UPARANT action on αvβ3 integrin and Rac‐1 was explored and its possible effects on ECM components including fibronectin, collagen I and collagen IV were considered as an indirect evidence of UPARANT role in the fibrotic process that was directly evaluated at the histological level. At the systemic level, the effects of UPARANT on standard renal parameters were investigated. In addition, we studied UPARANT role on vascular permeability (by evaluating occludin, zonula occludens [ZO]‐1, vascular endothelial growth factor [VEGF] and AQP2) and renal inflammation (by evaluating the inducible isoform of nitric oxide synthase [iNOS], intercellular adhesion molecule [ICAM]‐1, nuclear factor kappa‐light‐chain‐enhancer of activated B cells [NF‐kB], cAMP response element‐binding protein [CREB] and hypoxia‐inducible factor [HIF]‐1). Finally, we assessed the effects of UPARANT on DN‐associated morphological alterations of the glomerulus including a transmission electron microscopy (TEM) evaluation of the filtration barrier.

## 2 MATERIALS AND METHODS

### 2.1 Animals and treatment

Eighty‐four male Sprague‐Dawley rats (150‐200 g) were obtained from Envigo RMS, Italia (S. Pietro al Natisone, Italy). Of them, 63 received a single i.p. injection of 65 mg/kg STZ (Sigma‐Aldrich, St. Louis, MO, USA) in a citrate buffer solution (0.1 mol/L citric acid and 0.2 mol/L sodium phosphate, pH 4.5). Twenty‐one age‐matched rats received an equivalent volume of the citrate buffer solution (from now on referred as controls). Three days after STZ injection, blood glucose levels were measured. Animals with a plasma glucose >350 mg/dL were considered diabetic and were used for experimentation. Bodyweights and blood glucose levels were recorded once a week after the induction of diabetes. Four weeks after diabetes induction, three control or three STZ rats were used for the pharmacokinetics (PK) and the tissue distribution study. To this aim, rats received a single dose of UPARANT succinate dissolved in PBS (vehicle) at 20 mg/kg via subcutaneous injection according to a previous study.^22^ To investigate a possible therapeutic role of UPARANT, the drug was administered at 1 or 8 mg/kg daily for 5 days in 15 rats for each concentration used. Fifteen rats were left untreated while 15 rats were vehicle‐treated. In all experiments, no differences were observed between untreated and vehicle‐treated rats. UPARANT dose and regimen were in line with those used in previous studies.^21^, ^22^ The rats were kept individually in metabolic cages for 24 hours to collect urine for the measurement of urine output. Systolic blood pressure was measured by Tail‐cuff Blood Pressure System (IITC MRBP system Life Science, CA, USA). Rats were killed with 65 mg/kg pentobarbital. Blood was collected in EDTA tubes to then isolate plasma by centrifugation and kidneys were removed. Some of them were decapsulated and stored at −80°C until processing for protein extraction. Additional kidneys were fixed with 4% paraformaldehyde in PBS at 4°C and cryopreserved in 30% sucrose for immunohistochemistry or histological evaluation. Finally, the cortical part of some kidneys was cut and fixed in 4% glutaraldehyde and 1% osmium tetroxide for TEM evaluation. Procedures involving animals were carried out in compliance with the Italian guidelines for animal care (DL 26/14) and the European Communities Council Directive (2010/63/UE). Procedures were approved by the Ethical Committee in Animal Experiments of the University of Pisa.

### 2.2 Pharmacokinetics study and UPARANT tissue distribution

PK and UPARANT tissue distribution were as previously described.^21^ Briefly, for plasma PK, a volume of 0.2 mL blood was extracted at fixed time intervals (0.25, 0.5, 1, 2, 4, 6, 8, 12, 16 and 24 hours after dose) from the femoral vein. Blood was centrifuged (12 000 *g* for 20 minutes) and plasma was treated with 1% formic acid in methanol. Samples were stored at −80°C. The animals used for the PK study were killed at 24 hours. Kidneys and livers were dissected and processed with 1% formic acid in methanol. The concentration of UPARANT was evaluated via LC‐MS/MS.

### 2.3 Evaluation of albuminuria, creatininuria, plasma creatinine and blood urea nitrogen

Urine albumin was measured with the Albumin rat ELISA kit (Abcam, Cambridge, UK). Urine and plasma creatinine were measured with the Creatinine assay kit (Abcam). Blood urea nitrogen (BUN) was evaluated with the BUN colorimetric detection kit (Thermo Fisher Scientific, Waltham, MA, USA).

### 2.4 Western blotting

Whole kidneys homogenized using RIPA buffer supplemented with a cocktail of protease and phosphatase inhibitors were processed for protein extraction. They were used to investigate the effect of UPARANT on the uPAR pathway (uPA, uPAR and FPRs), αvβ3 integrin, the phosphorylated form of β3 integrin, Rac‐1, Plg, plasmin, MMPs (MMP‐2 and MMP‐9), markers of fibrosis (fibronectin, collagen I and collagen IV), markers of either vascular permeability (ZO‐1, occludin and VEGF) or inflammation (iNOS, ICAM‐1, phosphorylated and total forms of the p65 subunit of NF‐κB and of CREB, and the α subunit of HIF‐1 [HIF‐1α]). The effect of UPARANT on AQP2 was determined in samples of the medulla that was dissected from the renal cortex (3 medullas for each experimental condition). In all experiments, homogenates were centrifuged at 22 000 *g* for 15 minutes at 4°C. Protein concentration was evaluated using the Micro BCA method (Thermo Fisher Scientific). Equal amounts of proteins were separated by 4%‐20% SDS‐polyacrylamide gel electrophoresis gels (TGX Stain‐free precast gels; Bio‐Rad Laboratories, Hercules, CA, USA) and transferred onto nitrocellulose membrane using a Bio‐Rad Trans‐Blot Turbo System. The membranes were probed using the primary antibodies listed in Table S1. After the incubation with the appropriate horseradish‐peroxidase‐conjugated secondary antibody, bands were visualized using the Clarity Western ECL substrate with a ChemiDoc XP imaging system (Bio‐Rad Laboratories). Bands were quantified for densitometry using the Image Lab software (Bio‐Rad Laboratories) and normalized to β‐actin, NF‐κB or CREB, as appropriate.

### 2.5 Colorimetric assay

The activity of uPA and plasmin was measured using colorimetric assays. In particular, uPA activity was assayed using the uPA Activity Assay Kit (Sigma‐Aldrich) while plasmin activity was assayed using Chromozym PL, a plasmin‐specific chromogenic substrate (Sigma‐Aldrich).

### 2.6 Fluorogenic assay

The activity of MMPs was assessed using a fluorogenic assay (InnoZyme Gelatinase [MMP‐2/MMP‐9] Activity Assay Kit; Millipore, Bedford, MA, USA). The assay uses a collagen‐like fluorogenic substrate that, cleaved by MMP‐2/MMP‐9, results in fluorescence increase. Fluorescence was measured at an excitation wavelength of 320 nm and an emission wavelength of 405 nm.

### 2.7 ELISA

The activity of Rac‐1 was measured using the Rac‐1 Activation Assay Kit (Cytoskeleton, Inc., Denver, CO, USA), a quantitative ELISA assay that recognizes the active GTP‐bound form of Rac‐1. Plasma levels of uPA, (s)uPAR, Plg and MMPs were measured using commercially available ELISA kits (MyBioSource, San Diego, CA, USA for uPA; Lifescience Market, Hong Kong for (s)uPAR; Abcam for Plg and MMP‐2; LSBio, Seattle, WA, USA for MMP‐9).

### 2.8 Quantitative real‐time PCR

Kidneys were collected and placed in ice‐cold PBS (pH 7.4) and glomeruli were isolated from the cortical area through the passage to three consecutive cell strainers (200, 100 and 70 μm; pluriStrainer set 3, pluriSelect, Leipzig, Germany). The glomeruli retained on the 100 and 70 μm cell strainers were washed, assessed under a light microscope and used for quantitative real‐time PCR (qPCR) analysis. qPCR experiments were performed using three independent samples. Total RNA was extracted using RNeasy Mini Kit (Qiagen, Valencia, CA, USA). First‐strand cDNA was generated from 1 μg of total RNA (QuantiTect Reverse Transcription Kit; Qiagen). qPCR amplification was performed with SsoAdvanced Universal SYBR Green Supermix (Bio‐Rad Laboratories) on a CFX Connect Real‐Time PCR detection system and software CFX manager (Bio‐Rad Laboratories). qPCR primer sets for FPRs were chosen to hybridize to unique regions of the appropriate gene sequence. Their sequences were as follows: FPR1 forward 5′‐GTTTCCGCATGAAACGCACT‐3′; FPR1 reverse 5′‐CATGACCAGGCTGACGATGT‐3′; FPR2 forward 5′‐GCTTCACAATGCCCATGTCC‐3′; FPR2 reverse 5′‐ACTCGTAAGGGACGACTGGA‐3′; FPR3 forward 5′‐TCCCTTTCAACTGGTTGCCC‐3′; FPR3 reverse 5′‐GCCAATGAGTTGGTTGGCATA‐3′; Rpl13a forward 5′‐GGATCCCTCCACCCTATGACA‐3′; Rpl13a reverse 5′‐CTGGTACTTCCACCCGACCTC‐3′. Amplification efficiency was near 100% for each primer pair (Opticon Monitor 3 software; Bio‐Rad Laboratories). Target genes were assayed concurrently with Rpl13a, a gene encoding for ribosomal protein L13A. Samples were compared using the relative threshold cycle (Ct Method). The increase or decrease (fold change) was determined relative to control mice after normalization to Rpl13a. All reactions were performed in triplicate.

### 2.9 Histological evaluation

Kidney tissue samples were collected, fixed in 4% paraformaldehyde for 48 hours and embedded in paraffin. Five‐μm thick sections were dewaxed and stained with Masson's trichrome stain to detect fibrosis or periodic acid‐Schiff (PAS) to outline glomerular structure. Images were obtained by analysing a minimum of 15 glomerular sections from each group (five sections/animal) with a light microscope (Ni‐E, Nikon Europe, Amsterdam, The Netherlands). Glomerular and mesangial areas were evaluated using the Image J software (NIH, Bethesda, MD, USA).

### 2.10 Evans blue dye leakage

UPARANT effects on vascular permeability were determined by quantifying Evans blue dye leakage extravasation. Evans blue dye was dissolved in normal saline (20 mg/mL) and filtered. Anaesthetized rats were injected with Evans blue dye through the femoral vein at 20 mg/kg. Sixty minutes later, each rat was perfused through the left cardiac ventricle with 15 mL of heparinized saline (4 U/mL) under constant peristaltic flow (10 mL/min) to purge out the circulating dye. Then, the kidneys were harvested, dissected and weighed. The Evans blue dye was extracted with formamide overnight at 65°C and read at 620 nm using a plate reader (Microplate Reader 680 XR; Bio‐Rad Laboratories).

### 2.11 Immunofluorescence confocal analysis

Rat kidneys were fixed overnight with 4% paraformaldehyde at 4°C, cryopreserved in 30% sucrose for 24 hours, and embedded in optimal cutting temperature medium. Thin transverse cryosections (4 μm) were placed on Superfrost/Plus Microscope Slides (Thermo Fisher Scientific). The sections were incubated with a rabbit polyclonal antibody directed to AQP2 (1:1000 dilution)^23^ and then with an AlexaFluor488‐conjugated secondary antibody (Life Technologies, Carlsbad, CA, USA). Confocal images were obtained with a confocal laser‐scanning microscope (TSC‐SP2; Leica, Wetzlar, Germany).

### 2.12 Transmission electron microscopy

The cortical part of the kidneys was processed according to standardized procedures for electron microscopy. The sample were cut into 1 mm^3^ pieces, fixed in 4% glutaraldehyde (phosphate buffered, pH 7.2) and 1% osmium tetroxide and embedded in Epon 812 (Sigma‐Aldrich). After ultrathin sectioning, the samples were poststained with uranyLess TEM staining solution and examined under a JEM 1010 electron microscope (Jeol, Tokyo, Japan) at 80 kV. A minimum of 10 glomeruli were analysed from each group (three rats for each experimental condition) to evaluate the ultrastructural alterations of the glomerular filtration barrier.

### 2.13 Data analysis

Statistical significance was evaluated using one‐way ANOVA followed by Newman‐Keuls’ multiple comparison posttest. The results are expressed as mean ± SEM or mean ± SD of three independent measurements (Prism 5; GraphPad Software, San Diego, CA, USA). Differences with *P* < 0.05 were considered significant.

## 3 RESULTS

### 3.1 Plasma, renal and liver levels of UPARANT

**Figure 1:**
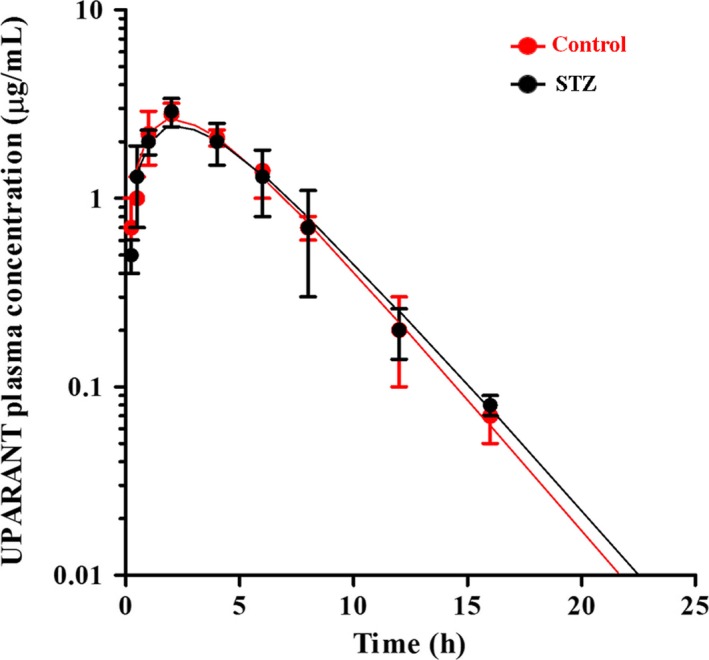
Average plasma concentrations of UPARANT after subcutaneous administration (20 mg/kg) to control (red dots and line) or STZ rats (black dots and line). Continuous lines correspond to the best fit of the equation C_t_ = C_0_(e^−kelim∙t^ − e^−kabs∙t^) where k_elim_ and k_abs_ correspond to the kinetic constants of the elimination and absorption phase, respectively. Values are expressed as means ± SD

Subcutaneously administered UPARANT rapidly appeared in the plasma being quantifiable at 0.25 hour, reached the C_max_ at 2.3 hours, and declined following a monophasic profile. UPARANT was still detectable at 24 hours reaching values that, although below the lower limit of quantification, were statistically different from the blank. These values are not represented in Figure 1 and were not considered in evaluating PK parameters in agreement with the FDA Guidance on Bioanalytical Method Validation.^24^ UPARANT was cleared from the plasma with a t_1/2_ of 2.2 hours in the elimination phase. Control values were in line with a previous work.^21^ No differences were found between control and STZ rats. Additional PK parameters as evaluated using a two‐phase model equation are summarized in Table S2. In the kidney of control rats, at 24 hours postdosing, the concentration of UPARANT was 2.2‐fold higher (*P* < 0.001; 45.9 ± 3.6 μg/g) than in the liver (21.1 ± 2.4 μg/g). In STZ rats, the liver concentration of UPARANT (20.4 ± 7.5 μg/g) did not differ from that in controls, whereas the UPARANT concentration in the diabetic kidney (28.2 ± 4.3 μg/g) was 1.6‐fold lower than in controls (*P* < 0.01).

### 3.2 The uPA/uPAR/FPR system

**Figure 2:**
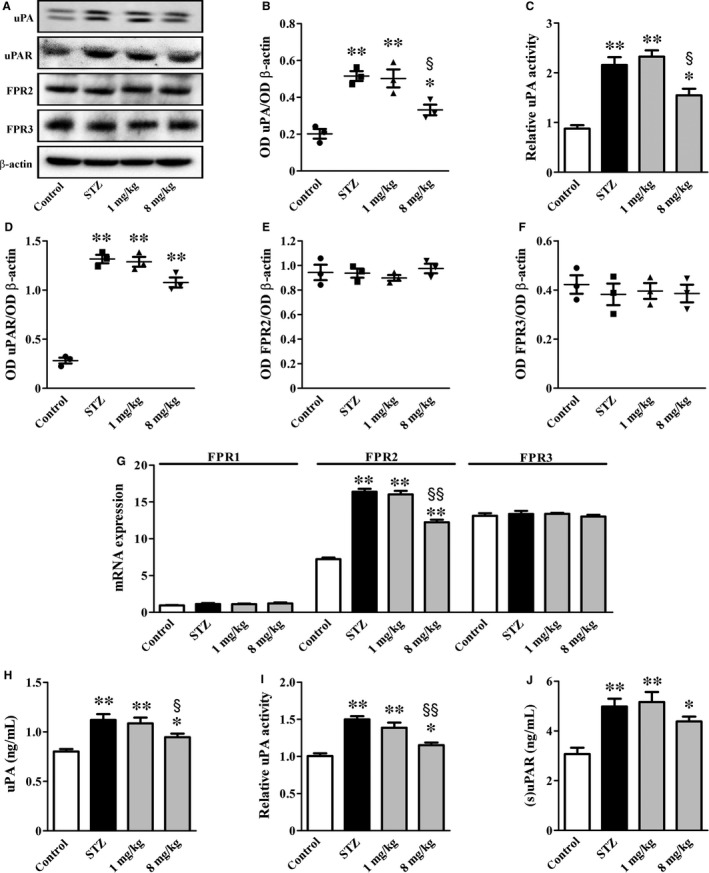
UPARANT effects on the uPA/uPAR/FPR system. (A) Representative blots showing protein levels of uPA, uPAR, FPR2 and FPR3 in kidney extracts. β‐actin was used as the loading control. (B, D‐F) Densitometric analysis showing that UPARANT at 1 mg/kg was ineffective on up‐regulated levels of uPA and uPAR, whereas at 8 mg/kg reduced uPA without affecting uPAR. (C) Up‐regulated uPA activity in kidney extracts was unaffected by UPARANT at 1 mg/kg, but was decreased by UPARANT at 8 mg/kg. (G) FPR transcripts in isolated glomeruli showing that UPARANT at 8 mg/kg, but not at 1 mg/kg, effectively reduced up‐regulated levels of FPR2 messengers. (H‐J) Up‐regulated uPA levels and activity in the plasma were reduced by UPARANT at 8 mg/kg, but not at 1 mg/kg. Up‐regulated (s)uPAR was unaffected at any drug concentration (*P < 0.05 and **P < 0.001 vs control; ^§^ *P* < 0.05 and ^§§^ *P* < 0.01 vs STZ; one‐way ANOVA followed by Newman‐Keuls’ multiple comparison posttest). Data are presented as scatter plots (B, D‐F) or histograms (C, G‐J). Each plot or histogram represents the mean ± SEM of data from three (B‐G) or seven (H‐J) independent samples

Representative blots of Figure 2A are indicative of the uPA/uPAR/FPR system activation in kidney extracts. As shown by Figure 2B‐F, high glucose enhanced renal levels of uPA and uPAR as well as uPA activity by about 2.6‐, 4.7‐ and 2.5‐fold (*P* < 0.001; Figure 2B‐D), while not affecting FPRs (Figure 2E and F). UPARANT at 1 mg/kg was ineffective on the uPAR pathway, whereas at 8 mg/kg reduced uPA levels and activity by 1.6‐ and 1.4‐fold (*P* < 0.05) without affecting uPAR levels. In isolated glomeruli, high glucose increased FPR2 transcript (2.3‐fold; *P* < 0.001) that was indeed reduced by UPARANT at 8 mg/kg (1.3‐fold; *P* < 0.01; Figure 2G). In the plasma, high glucose increased uPA levels and activity as well as (s)uPAR by 1.4‐, 1.5‐ and 1.6‐fold (*P* < 0.001, Figure 2H‐J). Both uPA levels and activity, but not (s)uPAR, were reduced by UPARANT at 8 mg/kg (1.2‐ and 1.3‐fold; *P* < 0.01).

### 3.3 αvβ3 integrin and Rac‐1 expression and activity

**Figure 3:**
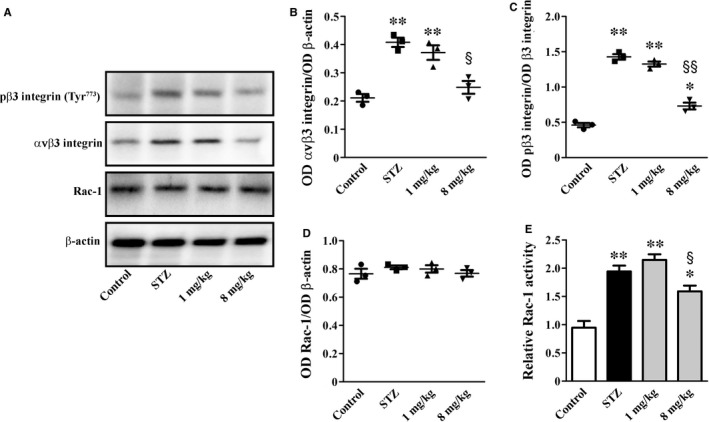
UPARANT effects on αvβ3 integrin and Rac‐1. (A) Representative blots showing protein levels of pβ3 integrin (Tyr^773^), αvβ3 integrin and Rac‐1. β‐actin was used as the loading control. (B‐D) Densitometric analysis showing that UPARANT at 8 mg/kg, but not at 1 mg/kg, reduced up‐regulated levels of pβ3 integrin (Tyr^773^) and αvβ3 integrin without affecting Rac‐1 levels. (E) Up‐regulated Rac‐1 activity was unaffected by UPARANT at 1 mg/kg, but was decreased by UPARANT at 8 mg/kg (*P < 0.05 and **P < 0.001 vs control; ^§^ *P* < 0.01 and ^§§^ *P* < 0.001 vs STZ; one‐way ANOVA followed by Newman‐Keuls’ multiple comparison posttest). Data are presented as scatter plots (B‐D) or histograms (E). Each plot or histogram represents the mean ± SEM of data from three independent samples

Representative blots of Figure 3A are indicative of the αvβ3 integrin signalling pathway. As shown in Figure 3B‐D, high glucose increased αvβ3 integrin levels and β3 integrin phosphorylation by 1.9‐ and 3.1‐fold (*P* < 0.001), while not affecting Rac‐1 levels. Rac‐1 activity increased by 2.1‐fold (*P* < 0.001; Figure 3E). UPARANT at 8 mg/kg, but not at 1 mg/kg, reduced αvβ3 integrin levels and β3 integrin phosphorylation as well as Rac‐1 activity by 1.6‐, 2.0‐ and 1.2‐fold (*P* < 0.01).

### 3.4 Fibrotic process

**Figure 4:**
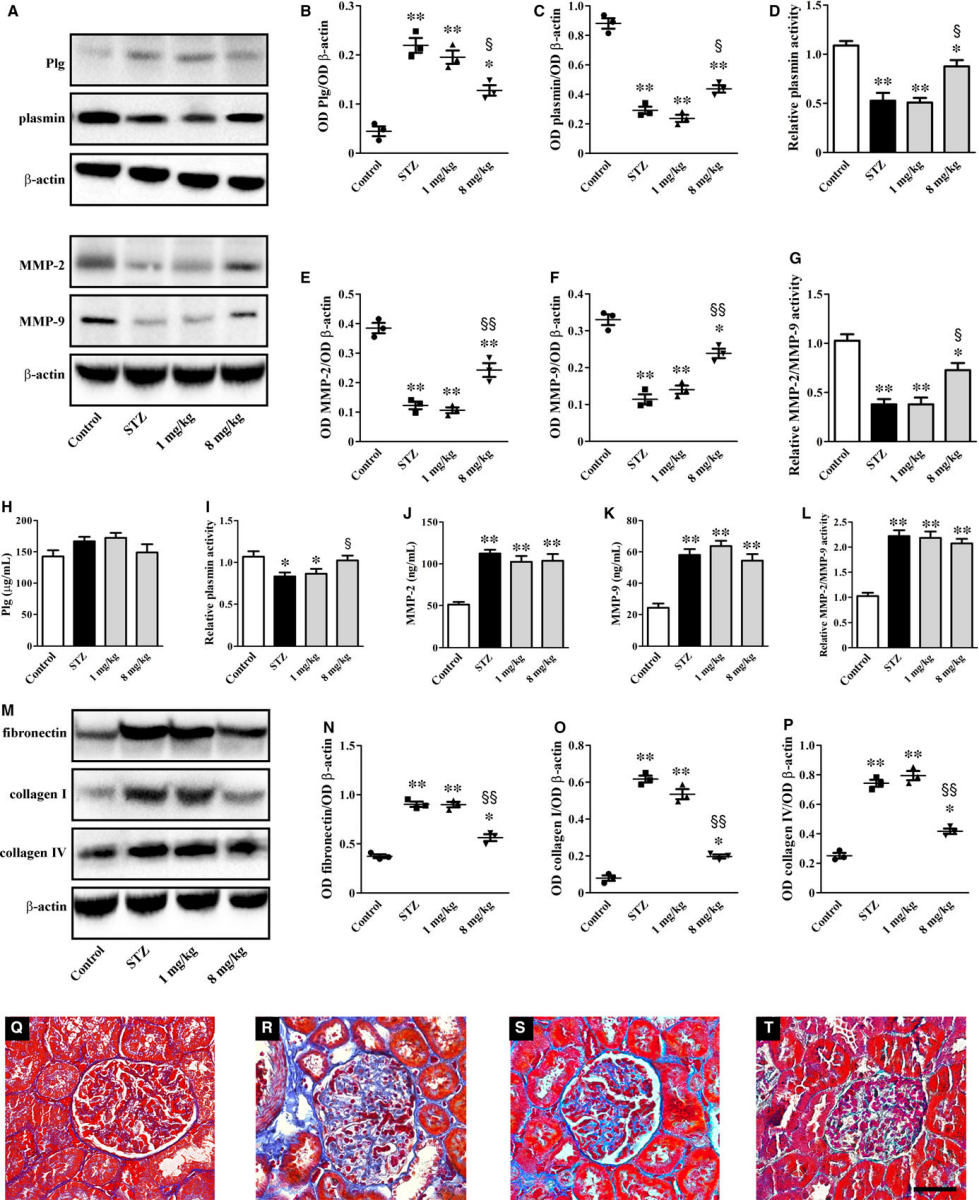
UPARANT effects on renal fibrosis. (A) Representative blots showing protein levels of Plg, plasmin, MMP‐2 and MMP‐9 in kidney extracts. β‐actin was used as the loading control. (B, C, E, F) Densitometric analysis showing that UPARANT at 8 mg/kg, but not at 1 mg/kg, almost recovered dysregulated levels of Plg, plasmin, MMP‐2 and MMP‐9. (D, G) Down‐regulated activity of both plasmin and MMPs was unaffected by UPARANT at 1 mg/kg, but was increased by UPARANT at 8 mg/kg. (H‐L) In the plasma, Plg levels, plasmin activity, MMP‐2 and MMP‐9 levels and their activity were unaffected by UPARANT with the exception of plamin activity that was recovered by UPARANT at 8 mg/kg. (M) Representative blots showing protein levels of ECM components including fibronectin, collagen I and collagen IV. β‐actin was used as the loading control. (N‐P) Densitometric analysis showing that UPARANT at 8 mg/kg, but not at 1 mg/kg, reduced up‐regulated levels of ECM components (*P < 0.01 and **P < 0.001 vs control; ^§^ *P* < 0.01 and ^§§^ *P* < 0.001 vs STZ; one‐way ANOVA followed by Newman‐Keuls’ multiple comparison posttest). Data are presented as scatter plots (B, C, E, F, N‐P) or histograms (D, G, H‐L). Each plot or column represents the mean ± SEM of data from three (B‐G, N‐P) or seven (H‐L) independent samples. (Q‐T) Histological assessment of renal fibrosis in control (Q) and STZ rats either untreated (R) or treated with UPARANT at 1 mg/kg (S) or 8 mg/kg (T). Representative photomicrographs from Masson's Trichrome staining of sections that are representative of three animals/group showing that increased glomerular fibrotic areas (stained in blue) were reduced by UPARANT at 8 mg/kg, but not at 1 mg/kg. Scale bar: 50 μm

Representative blots of Figure 4A are indicative of renal levels of secreted proteases that are known to mediate ECM remodelling. As shown in Figure 4B‐G, high glucose increased Plg by 4.9‐fold (*P* < 0.001), but decreased either plasmin levels and activity (3.0‐ and 2.1‐fold; *P* < 0.001) or MMP‐2/MMP‐9 levels and activity (3.1‐, 2.9‐ and 2.7‐fold; *P* < 0.001). UPARANT at 1 mg/kg was ineffective on secreted proteases, while at 8 mg/kg reduced Plg levels by 1.7‐fold (*P* < 0.01), increased either plasmin levels and activity by 1.5‐ and 1.7‐fold (*P* < 0.01) or MMP‐2/MMP‐9 levels and activity by 2.0‐, 1.7‐ and 1.9‐fold (*P* < 0.01). Levels and activity of secreted proteases were also assessed in the plasma (Figure 4H‐L). Plg levels were not affected by hyperglycaemia that, on the contrary, reduced plasmin activity by 1.3‐fold (*P* < 0.01) and increased MMP‐2/MMP‐9 levels and activity by 2.2‐, 2.4‐ and 2.2‐fold (*P* < 0.001). UPARANT at 8 mg/kg did not affect Plg levels, but increased plasmin activity by 1.3‐fold (*P* < 0.01) without influencing MMP‐2/MMP‐9 levels and activity. Representative blots of Figure 4M are indicative of protein levels of ECM components. As shown in Figure 4N‐P, high glucose increased fibronectin, collagen I and collagen IV by 2.4‐, 7.7‐ and 2.9‐fold (*P* < 0.001). UPARANT at 8 mg/kg reduced fibronectin, collagen I and collagen IV by about 1.6‐, 3.1‐ and 1.8‐fold (*P* < 0.001) as an indirect evidence of its ameliorative effect on fibrotic process. In agreement with these data, the histological assessment of renal fibrosis by Masson's Trichrome staining showed that when comparing kidney sections from STZ rats with those from control rats, the glomerular fibrotic areas (stained in blue) were significantly increased in STZ rats. Of note, they were decreased by UPARANT at 8 mg/kg, but not at 1 mg/kg (Figure 4Q‐T).

### 3.5 Standard renal parameters

As shown in Table 1, the bodyweight of STZ rats was 1.5‐fold lower than in controls (*P* < 0.001). Both kidney weight/bodyweight and blood glucose significantly increased by 1.6‐ and 4.2‐fold (*P* < 0.001). Treatment with UPARANT at 1 or 8 mg/kg did not affect these parameters. STZ rats also showed increased urine output, urine albumin, urine creatinine, albumin to creatinine ratio, plasma creatinine, creatinine clearance and BUN by about 10.5, 3.3‐, 1.3‐, 3.8‐, 2.2‐, 2.1‐ and 3.1‐fold, respectively (*P* < 0.001). UPARANT at 1 mg/kg did not affect these parameters, whereas at 8 mg/kg significantly reduced them by 1.7‐, 1.8‐, 1.3‐, 2.3‐, 1.4‐, 1.3‐ and 1.7‐fold, respectively (*P* < 0.001). No differences in systolic blood pressure were observed among the experimental groups.

**Table 1:** Physiological and renal parameters

	Control	STZ	STZ + UPARANT, 1 mg/kg	STZ + UPARANT, 8 mg/kg
Bodyweight (g)	373.7 ± 2.7	245.2 ± 2.6*	246.3 ± 4.0*	248.3 ± 3.0*
Kidney weight/Bodyweight (mg/g)	2.64 ± 0.12	4.26 ± 0.09*	4.13 ± 0.08*	4.03 ± 0.08*
Blood glucose (mg/dL)	127.2 ± 5.5	529.8 ± 18.3*	539.3 ± 20.3*	563.5 ± 11.7*
Urine output (mL)	15.6 ± 0.9	163.1 ± 7.2*	158.7 ± 6.2*	94.4 ± 1.9* ^,^ †
Urine albumin (mg/24 h)	0.628 ± 0.013	2.088 ± 0.047*	2.080 ± 0.079*	1.178 ± 0.039* ^,^ †
Urine creatinine (mg/24 h)	13.43 ± 0.25	17.40 ± 0.41*	17.27 ± 0.45*	13.36 ± 0.26†
Albumin to creatinine ratio	0.041 ± 0.001	0.154 ± 0.005*	0.153 ± 0.003*	0.066 ± 0.002* ^,^ †
Plasma creatinine (mg/mL)	0.430 ± 0.017	0.937 ± 0.043*	0.925 ± 0.042*	0.653 ± 0.048* ^,^ †
Creatinine clearance (μL/min/g)	3.521 ± 0.177	7.560 ± 0.290*	7.366 ± 0.382*	5.976 ± 0.313* ^,^ †
Blood urea nitrogen (mmol/L)	7.71 ± 0.20	23.82 ± 0.50*	23.25 ± 0.41*	13.85 ± 0.40* ^,^ †
Systolic blood pressure (mmHg)	125.4 ± 5.1	127.3 ± 1.9	130.3 ± 3.4	127.4 ± 3.9

*P < 0.001 vs control; ^†^ *P* < 0.001 vs STZ (one‐way ANOVA followed by Newman‐Keuls’ multiple comparison posttest).

### 3.6 Vascular permeability

**Figure 5:**
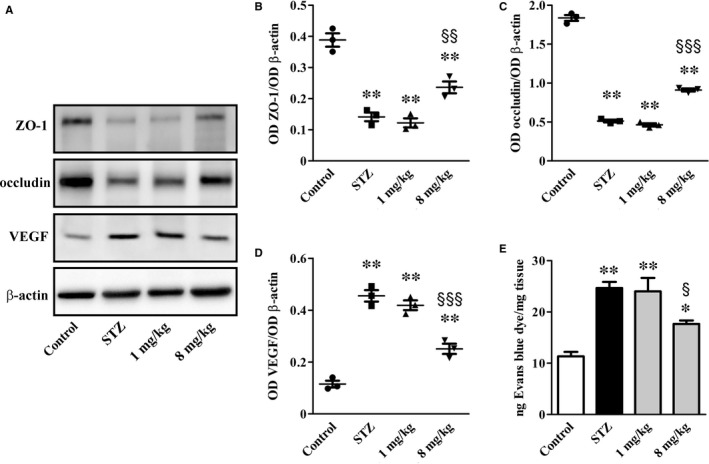
UPARANT effects on vascular permeability markers and Evans blue dye leakage. (A) Representative blots showing protein levels of ZO‐1, occludin and VEGF. β‐actin was used as the loading control. (B‐D) Densitometric analysis showing that UPARANT at 8 mg/kg, but not at 1 mg/kg, increased ZO‐1 and occludin while decreased VEGF. (E) Quantitative evaluation of Evans blue extravasation. Increased Evans blue dye that was almost recovered by UPARANT at 8 mg/kg but not at 1 mg/kg (*P < 0.01 and **P < 0.001 vs control; ^§^ *P* < 0.05, ^§§^ *P* < 0.01 and ^§§§^ *P* < 0.001 vs STZ; one‐way ANOVA followed by Newman‐Keuls’ multiple comparison posttest). Data are presented as scatter plots (B‐D) or histograms (E). Each plot or column represents the mean ± SEM of data from three independent samples

A prediction of the vascular permeability integrity was performed by analysing the expression levels of (a) ZO‐1 and occludin, two tight junction proteins participating in the glomerular filtration barrier and (b) the propermeability factor VEGF (Figure 5A). In STZ rats, ZO‐1 (Figure 5B) and occludin (Figure 5C) decreased by 2.7‐ and 3.6‐fold (*P* < 0.001) in agreement with previous studies,^25^ while VEGF increased (3.9‐fold, *P* < 0.001; Figure 5D) in line also with previous results.^26^ In addition, high glucose caused plasma extravasation as determined by an increased amount of Evan's blue (2.2‐fold, *P* < 0.001; Figure 5E). UPARANT at 1 mg/kg resulted ineffective in the restoration of the expression levels of these markers, whereas at 8 mg/kg both increased ZO‐1 (1.7‐fold, *P* < 0.01) and occludin levels (1.8‐fold, *P* < 0.001), and decreased VEGF expression levels (1.8‐fold, *P* < 0.001) and plasma extravasation (1.4‐fold, *P* < 0.05) compared to untreated STZ rats.

**Figure 6:**
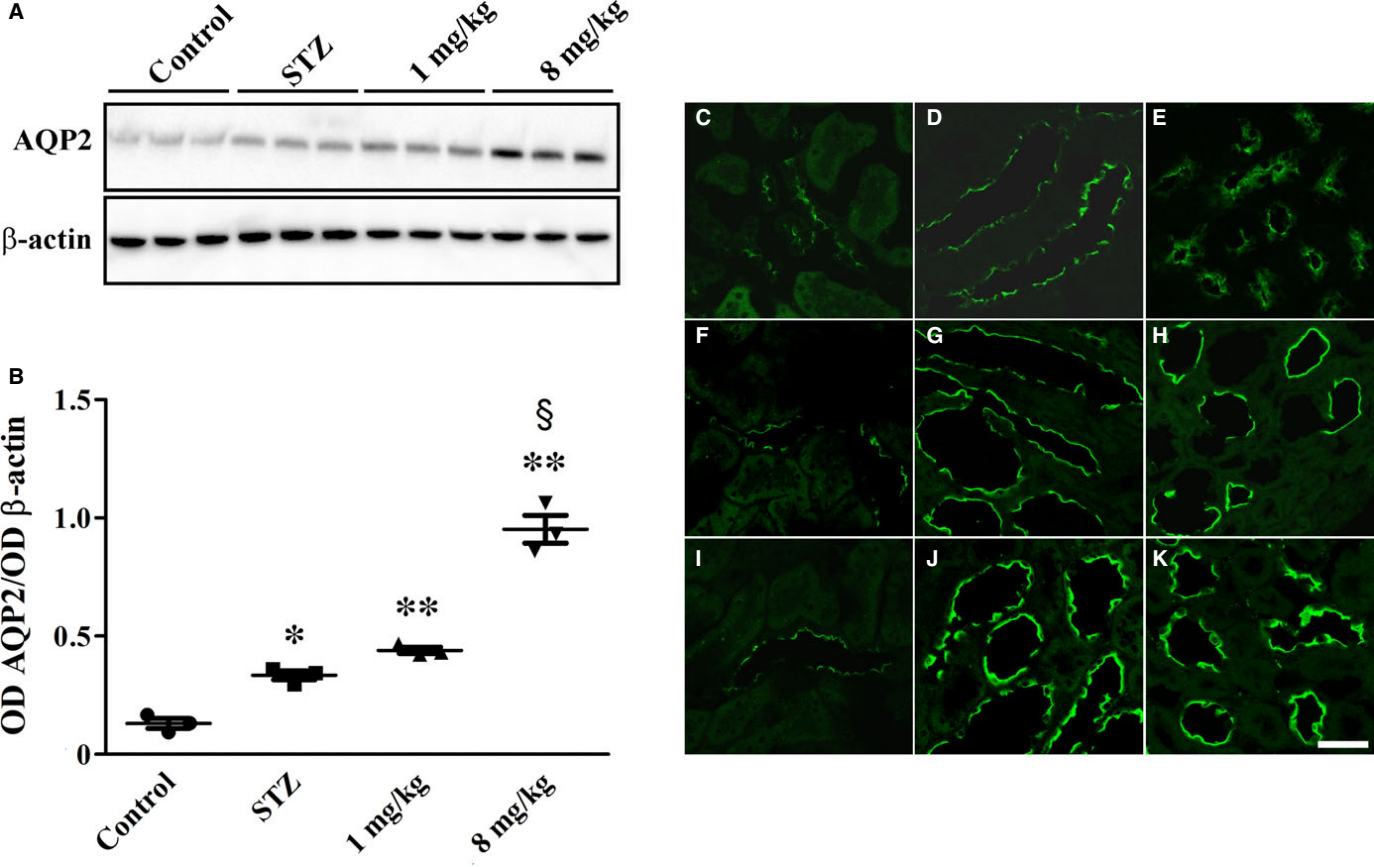
UPARANT effects on AQP2 expression and localization. (A) Representative blots showing protein levels of AQP2 in the kidney medulla. β‐actin was used as the loading control. (B) Densitometric analysis showing that UPARANT at 8 mg/kg increased high glucose‐induced AQP2 up‐regulation, while it was ineffective at 1 mg/kg (*P < 0.01 and **P < 0.001 vs control; ^§^ *P* < 0.001 vs STZ; one‐way ANOVA followed by Newman‐Keuls’ multiple comparison posttest). Data are presented as scatter plots. Each plot represents the mean ± SEM of data from three independent samples. (C‐K) AQP2 immunoreactivity in control (C‐E) and STZ rats either untreated (F‐H) or treated with UPARANT 8 mg/kg (I‐K). Representative photomicrographs from sections that are representative of three animals/group showing that AQP2 is expressed at the apical plasma membrane of cells lining the collecting ducts of the cortex (C) and the medulla (outer in D and inner in E). STZ did not modify AQP2 expression in the cortex (F), whereas increased the apical expression of AQP2 in the medulla (outer in G and inner in H). UPARANT at 8 mg/kg did not change AQP2 expression in the cortex (I), whereas additionally increased AQP2 apical expression in the medulla (outer in J and inner in K). Scale bar: 50 μm

Decreased plasma extravasation would result in decreased urine output to which UPARANT‐associated increase in AQP2 expression in the medulla may contribute (Figure 6A). As shown in Figure 6B and in line with previous findings,^4^ DN was associated with relative abundance of AQP2 (2.6‐fold, *P* < 0.01). UPARANT at 1 mg/kg did not influence AQP2 up‐regulation, whereas at 8 mg/kg it further increased AQP2 levels (2.8‐fold, *P* < 0.001). In addition, in control rats, AQP2 was localized to the apical plasma membrane of cells lining the collecting ducts in both the cortex and the medulla (Figure 6C‐E). No differences between control and STZ rats were found in the cortex (Figure 6F), while AQP2 apical expression was higher in the medulla of STZ rats (Figure 6G and H). UPARANT at 8 mg/kg did not influence AQP2 expression in the cortex (Figure 6I), while further increased AQP2 immunoreactivity in the medulla particularly at the apical membrane (Figure 6J and K).

### 3.7 Inflammatory markers

**Figure 7:**
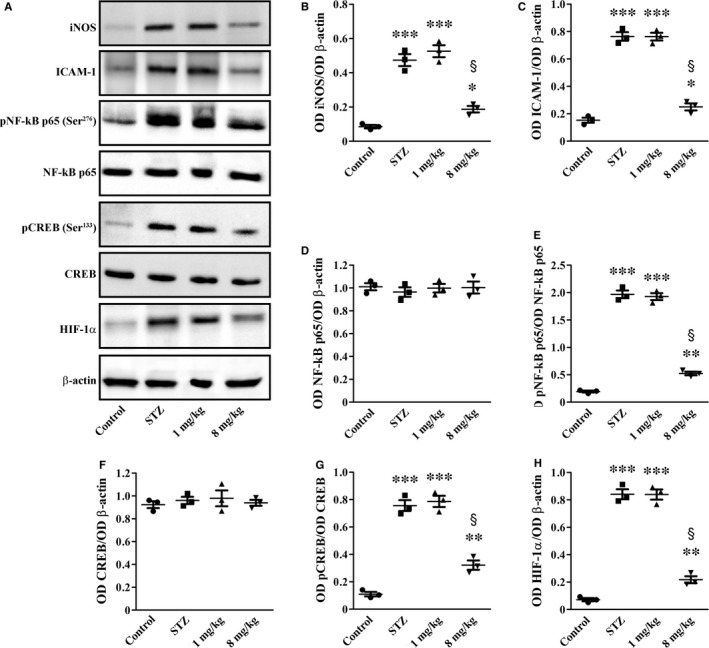
UPARANT effects on inflammatory markers. (A) Representative blots showing protein levels of iNOS, ICAM‐1, pNF‐kB p65 (Ser^276^), NF‐kB p65, pCREB (Ser^133^), CREB and HIF‐1α. β‐actin was used as the loading control. (B‐H) Densitometric analysis showing that UPARANT at 8 mg/kg, but not at 1 mg/kg, reduced up‐regulated levels of iNOS, ICAM‐1, pNF‐kB p65 (Ser^276^), pCREB (Ser^133^) and HIF‐1α (*P < 0.05, **P < 0.001 and ***P < 0.001 vs control; ^§^ *P* < 0.001 vs STZ; one‐way ANOVA followed by Newman‐Keuls’ multiple comparison posttest). Data are presented as scatter plots. Each plot represents the mean ± SEM of data from three independent samples

As shown in Figure 7 and in line with previous findings,^27^, ^28^, ^29^ the levels of iNOS, ICAM‐1 and HIF‐1α, as well as the phosphorylation of NF‐κB p65 at Ser^276^ and CREB at Ser^133^, were higher in STZ rats than in controls (5.0‐, 5.6‐, 11.7‐, 10.2‐ and 6.9‐fold, respectively; *P* < 0.001). Inflammatory markers were not affected by UPARANT at 1 mg/kg, whereas UPARANT at 8 mg/kg reduced iNOS, ICAM‐1, NF‐κB p65 phosphorylation, CREB phosphorylation and HIF‐1α by 3.0‐, 2.5‐, 3.8‐, 2.4‐ and 3.9‐fold, respectively (*P* < 0.001).

### 3.8 Pathological findings in the kidney

**Figure 8:**
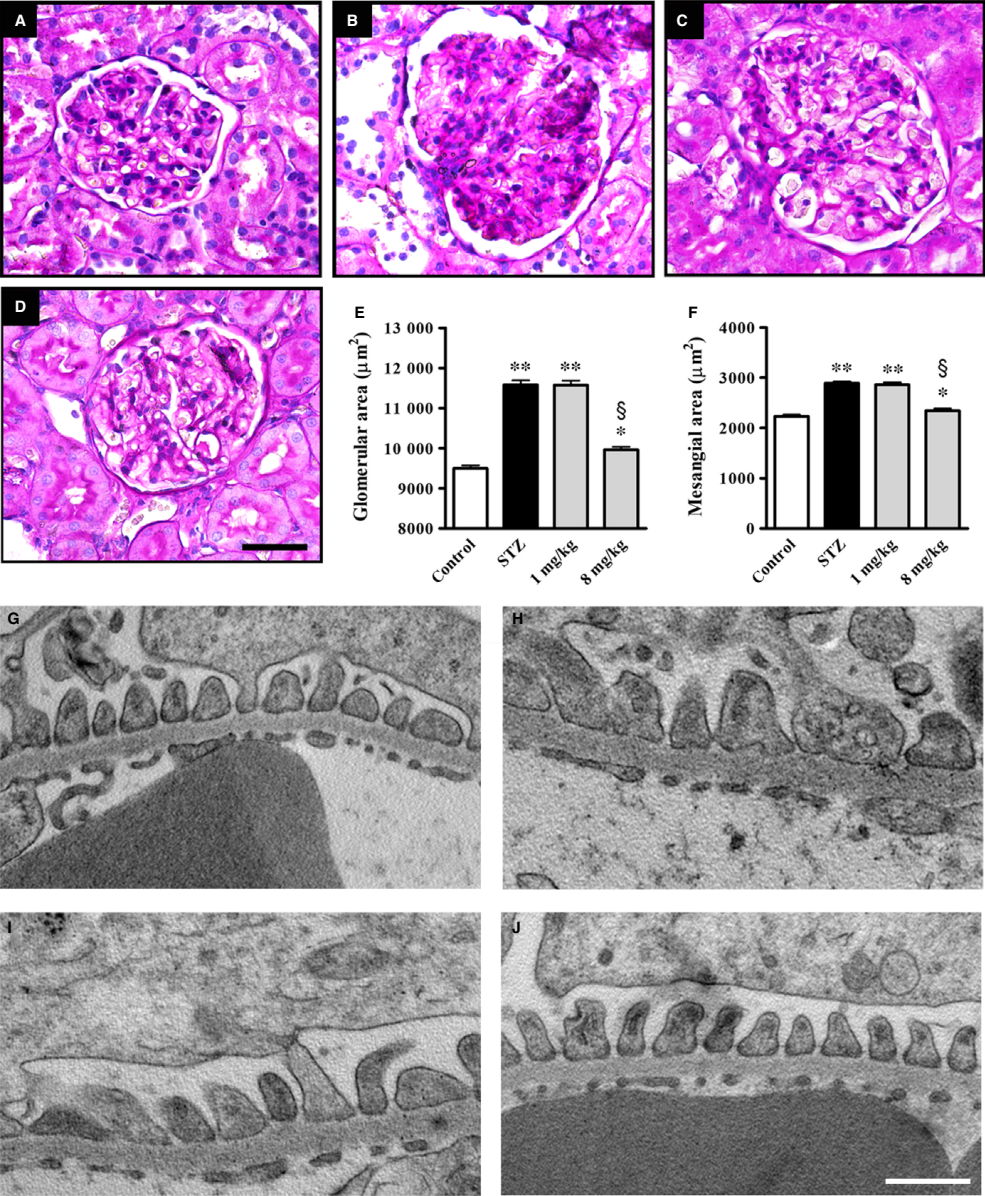
Effects of UPARANT on glomerular morphology and filtration barrier. (A‐D) Representative histological sections stained with periodic acid‐Schiff of kidneys from control (A) and STZ rats either untreated (B) or treated with UPARANT at 1 mg/kg (C) or 8 mg/kg (D). UPARANT at 1 mg/kg did not affect the glomerular morphology (C), whereas at 8 mg/kg attenuated glomerular hypertrophy and mesangial expansion (D). Scale bar: 50 μm. (E, F) Quantitative evaluation of glomerular hypertrophy (E) and increased mesangial area (F) confirmed qualitative assessment (*P < 0.05 and **P < 0.001 vs control; ^§^ *P* < 0.001 vs STZ; one‐way ANOVA followed by Newman‐Keuls’ multiple comparison posttest). Data are presented as histograms. Each column represents the mean ± SEM of data from 15 glomerular sections (five sections/animal, three animals/group). (G‐J) UPARANT effects on glomerular filtration barrier morphology. EM micrographs at 30 K magnification that are representative of three animals/group showing ultrathin podocyte sections from control (G) or STZ rats either untreated (H) or treated with UPARANT at 1 mg/kg (I) or 8 mg/kg (J). UPARANT at 8 mg/Kg, but not at 1 mg/kg, reduced high glucose‐induced thickening of the glomerular basement membrane and almost recovered foot process effacement. Scale bar: 500 nm

Figure 8A‐D shows the results of histological examination of kidney sections stained with PAS. The kidneys of control rats revealed normal glomeruli (Figure 8A), while those of STZ rats (Figure 8B) showed glomerular hypertrophy and increased mesangial area in agreement with previous results.^30^ Treatment with UPARANT at 1 mg/kg was ineffective (Figure 8C), whereas UPARANT at 8 mg/kg prevented these modifications (Figure 8D). As shown by the quantitative analysis, the glomerular area (Figure 8E) and the mesangial area (Figure 8F) were increased by 1.2‐ and 1.3‐fold (*P* < 0.001) as compared to controls. These parameters were reduced by UPARANT at 8 mg/kg by 1.1‐ and 1.2‐fold (*P* < 0.001), while no effects were observed after UPARANT at 1 mg/kg. The characteristic alterations of DN are well evident in the TEM representative images of Figure 8G‐J. In particular, their qualitative analysis demonstrates thickening of the glomerular basement membrane and loss of podocyte foot processes as shown by the increase in their base width and the reduction in their number. UPARANT at 1 mg/kg was ineffective, whereas at 8 mg/kg prevented the modifications of the glomerular filtration barrier by almost restoring the normal architecture.

## 4 DISCUSSION

This paper provided the first evidence that blocking the uPAR pathway with UPARANT, a small peptide with a predominant anti‐inflammatory action, can improve diabetic kidney lesion in rats. The efficacy of UPARANT in DN suggests a pivotal role of the uPAR system in the pathogenesis of diabetic complications, such as DN and DR that share similar pathogenesis mechanisms including dysregulated uPAR pathway. In this respect, the possible use of drugs targeting mechanisms common to DN and DR is of increasing interest to treat both diabetes complications at the same time. In addition, whether drugs regulating uPAR activation might be administered in association with hypoglycaemic drugs, then the possibility can be hypothesized that diabetes‐associated renal complications can be retarded or even abolished.

### 4.1 UPARANT delivery and administration regimen

As shown here, the plasma concentration of UPARANT is comparable in control and STZ rats in line with previous results^21^ and with the fact that systemically delivered drugs have been found to display comparable PK profiles in healthy and diabetic rats.^31^, ^32^ Tissue UPARANT concentration found here reflects a major role of the kidney in regulating drug distribution and metabolism. The lower UPARANT concentration found in the diabetic kidney suggests that altered kidney function may affect the excretion of the compound that, although filtered at the glomerular level, may be reabsorbed by the tubules, thus shifting its elimination route. UPARANT dose and regimen used here are in line with those used in previous studies in which systemic treatment with the drug has been shown both effective and safe.^21^, ^22^ In particular, UPARANT dosage is almost 50% less than that used in the PK study and is in line with the UPARANT concentration measured in the kidney.

### 4.2 Recovery of the uPA/uPAR/FPR system

Increased activation of the uPAR pathway and increased (s)uPAR levels are indicative of uPA/uPAR contribution to a proteolytic cascade that has detrimental effects on glomerular cell permeability.^33^ In addition, FPR2 overexpression found here in isolated glomeruli is in line with FPR accumulation in models of impaired nephrogenesis in which FPR2, in particular, appears to participate to the fibrosis process.^34^, ^35^ As shown by the present results, UPARANT efficacy against diabetic kidney lesion is associated to decreased uPA accumulation and activity, thus presumably influencing ligand availability to its receptors and increasing drug efficacy more than if the drug acted at the postreceptor level only. In this line, there is evidence that reducing uPA‐uPAR interactions results in beneficial effects on kidney lesions.^36^, ^37^ As also shown here, UPARANT does not affect (s)uPAR up‐regulation in agreement with previous findings demonstrating that blockers of the renin angiotensin system commonly used to prevent or delay DN do not affect plasma (s)uPAR in diabetic patients.^38^ The additional finding that the drug does not influence uPAR in podocytes is in line with the possibility that UPARANT acts downstream uPAR by presumably blocking its binding to FPRs. In this respect, UPARANT has been designed to mimic the sequence through which uPAR not only interacts with FPRs thus competing with FPR ligands,^15^ but also binds vitronectin thus activating αvβ3 integrin^39^ that is indeed forced into an inactive state.^15^

### 4.3 Recovery of kidney lesions

Plg is mainly synthesized in the liver, circulates in the plasma and is activated to plasmin by a finely tuned balance between activators, including uPA, and inhibitors: a dysregulation of this system is reported in renal diseases, including DN, but the mechanisms through which it contributes to diabetic kidney lesions are not still completely understood.^9^ The present finding that UPARANT recovers, at least in part, plasmin activity is indicative of the possibility that the drug may reinstate the physiological balance between activators and inhibitors, thus reactivating the proteolytic cascade that leads to the degradation of ECM components. The main regulators of ECM degradation in the glomerulus are MMPs, being a balance between ECM synthesis and degradation a prerequisite to maintain glomerulus integrity. In particular, MMP‐2 and MMP‐9 are considered as the main enzymes degrading collagen IV, the major collagenous component of ECM constituting the architectural structure of glomerular basement membrane.^40^ Thus, high glucose‐induced reduction in renal MMPs as observed here, may be directly translated into altered ECM turnover and is consistent with the increased deposition of ECM components leading to glomerular damage and a decline in renal function.^41^ As also shown here, high glucose increases plasma levels and activity of MMP‐2/MMP‐9 in line with previous findings in diabetic patients with abnormal ECM metabolism.^42^ The present finding that at the renal level UPARANT counteracts high glucose‐associated dysregulation of MMPs is indicative of its major effects on ECM‐degrading proteases. This is in line with the fact that increased uPA/uPAR expression correlates well with ECM accumulation in STZ rats,^43^ while uPAR knockdown reduces MMPs expression in cultured kidney cells.^44^ The finding that UPARANT does not affect plasma MMPs suggests the predominance of local effects on MMPs‐expressing podocytes^45^ over systemic effects on secreted proteases also in line with previous studies using antioxidative compounds in the STZ model.^46^ As a consequence of the restored MMPs activity, UPARANT reduces renal fibrosis as indirectly evidenced by decreased levels of ECM components and directly observed by the marked reduction of Masson's staining. This is in line with previous findings demonstrating that drugs counteracting MMPs dysregulation are found to inhibit ECM accumulation, thus preventing glomerular damage in STZ rats.^47^, ^48^ To the best of our knowledge, this is the first report indicating that inhibiting the uPAR pathway restores MMPs, thus suggesting a possible mechanism through which UPARANT may act to maintain the structural and functional integrity of the glomerulus. As also observed here, the UPARANT‐induced recovery of impaired glomerular function seems to be the fundamental way to lower altered renal parameters. In particular, recovered proteinuria is in line with uPAR role in regulating the αvβ3 integrin/Rac‐1 pathway and suggests the possibility that uPAR interacts with αvβ3 integrin receptors to affect podocyte function.^10^ Indeed, as shown here, up‐regulated levels of αvβ3 integrin and increased Rac‐1 activity are recovered by UPARANT indicating a major role of uPAR (as well as (s)uPAR from circulation) to regulate the αvβ3 integrin/Rac‐1 pathway in podocytes. In this respect, induction of uPAR signalling in podocytes leads to foot process effacement and urinary protein loss via a mechanism that includes the activation of the αvβ3 integrin/Rac‐1 pathway.^36^ Thus, preventing uPAR/(s)uPAR interactions with αvβ3 integrin is likely to affect the activation of the αvβ3 integrin/Rac‐1 pathway and the uPAR/(s)uPAR‐mediated podocyte injury.^10^ Ameliorative effects upon the inhibition of the uPAR pathway are in agreement with the finding that uPAR down‐regulation restores the filtration barrier function in cultured podocytes^49^ and reduces proteinuria in lipopolysaccharide‐treated mice.^36^, ^37^ This is also in line with the present finding that UPARANT‐induced recovery of altered vascular permeability is established through restored levels of tight junction proteins and inhibition of VEGF production. These results together suggest an additional mechanism through which the uPAR pathway negatively affects glomerular filtration and are in line with previous findings demonstrating that deletion of various components of the uPA/uPAR system is protective against vascular changes in models of renal diseases.^9^ Recovered vascular permeability is associated with UPARANT‐induced increase of AQP2 in the medulla in which AQP2 seems to prevent excessive water loss through urine.^4^ In this respect, therapies that up‐regulate AQP2 expression in the inner medulla have been shown to prevent diabetic kidney disease.^50^ Additional effects of UPARANT include its potent anti‐inflammatory action. This is particularly interesting since inflammation has been shown to play a major role in diabetic kidney disease and the suboptimal efficacy of the current therapeutic strategies may depend on their limited impact on inflammatory processes.^51^ Flavonoids, for instance, have been shown to attenuate DN more efficiently than commercial antidiabetic drugs via suppressing the activation of NF‐κB and decreasing ICAM‐1 and iNOS.^52^, ^53^ As shown here, UPARANT inhibits up‐regulated levels of NF‐κB, which, in addition to activate gene transcription of inflammatory factors,^54^ promotes uPA transcription^55^ and regulates ECM production.^56^ In addition, NF‐κB regulation of ICAM‐1 positively correlates with nephropathy^57^ by influencing mesangial cell proliferation.^58^ Moreover, NF‐κB participates to iNOS accumulation^59^ and, together with CREB, regulates AQP2 gene transcription.^60^ Whether UPARANT‐induced inhibition of HIF‐1α accumulation ameliorates kidney disease remains unclear as to some extent the activation of HIF‐1, but not its inhibition, seems to exert a beneficial role in the progression of DN.^61^ Suppression of inflammatory processes and ameliorated renal fibrosis results in recovered renal morphology as shown here by the reduction of glomerular hypertrophy and mesangial area increase.^62^ Additional efficacy of UPARANT includes ameliorated renal filtration barrier as demonstrated by recovery from increased thickness of glomerular basement membrane and from loss of podocyte foot processes leading to proteinuria. Consistently, the observed reduction in the urine albumin level in UPARANT‐treated rats is congruent with UPARANT‐induced protection of the renal architecture.

## 5 CONCLUSION

Together, the present findings support the possibility that uPA/uPAR activation in response to high glucose participates in the mechanisms causing DN and provide evidence that the uPAR pathway is a promising target for the development of novel multitarget drugs in the treatment of diabetic kidney disease. By acting on multiple pathways involved in DN pathogenesis, UPARANT constitutes a promising strategy to cure the diseased kidney although the extrapolation of these experimental findings to the clinic is not straightforward.

## CONFLICT OF INTEREST

Ma.C. received a grant from Kaleyde Pharmaceuticals AG. M.D.R. and Vi.P. are the holders of UPARANT patent. M.D.M., Va.P., Mo.C., G.P., M.S., A.P. and P.B. declare no conflicts of interest.

## AUTHOR CONTRIBUTION

MDM and MaC performed the experiments, analysed the data and contributed to writing the manuscript; VaP performed the experiments; MoC and GP performed the experiments and analysed the data; AP performed the electron microscopy; MDR and ViP designed the research study, interpreted the data and contributed to writing the manuscript; MS contributed to writing the manuscript; PB designed the research study, interpreted the data and wrote the manuscript.

## References

[1] Tesch GH , Allen TJ . Rodent models of streptozotocin‐induced diabetic nephropathy. Nephrology. 2007;12:261‐266. 17498121 10.1111/j.1440-1797.2007.00796.x

[2] Peng T , Chang X , Wang J , et al. Protective effects of tacrolimus on podocytes in early diabetic nephropathy in rats. Mol Med Rep. 2017;15:3172‐3178. 28339051 10.3892/mmr.2017.6354

[3] Bhattacharjee N , Barma S , Konwar N , et al. Mechanistic insight of diabetic nephropathy and its pharmacotherapeutic targets: an update. Eur J Pharmacol. 2016;791:8‐24. 27568833 10.1016/j.ejphar.2016.08.022

[4] Kim D , Sands JM , Klein JD . Changes in renal medullary transport proteins during uncontrolled diabetes mellitus in rats. Am J Physiol Renal Physiol. 2003;285:F303‐F309. 12697581 10.1152/ajprenal.00438.2002

[5] Fenton RA , Pedersen CN , Moeller HB . New insights into regulated aquaporin‐2 function. Curr Opin Nephrol Hypertens. 2013;22:551‐558. 23852332 10.1097/MNH.0b013e328364000d

[6] Liu J , Wei Q , Guo C , et al. Hypoxia, HIF, and associated signaling networks in chronic kidney disease. Int J Mol Sci. 2017;18:E0950. 28468297 10.3390/ijms18050950 PMC5454863

[7] Sanchez AP , Sharma K . Transcription factors in the pathogenesis of diabetic nephropathy. Expert Rev Mol Med. 2009;11:e13. 19397838 10.1017/S1462399409001057

[8] Prevete N , Liotti F , Marone G , et al. Formyl peptide receptors at the interface of inflammation, angiogenesis and tumor growth. Pharmacol Res. 2015;102:184‐191. 26466865 10.1016/j.phrs.2015.09.017

[9] Svenningsen P , Hinrichs GR , Zachar R , et al. Physiology and pathophysiology of the plasminogen system in the kidney. Pflugers Arch. 2017;469:1415‐1423. 28656379 10.1007/s00424-017-2014-y

[10] Dande RR , Peev V , Altintas MM , Reiser J . Soluble urokinase receptor and the kidney response in diabetes mellitus. J Diabetes Res. 2017;2017:3232848. 28596971 10.1155/2017/3232848 PMC5449757

[11] Hall SS . Omen in the blood. Science. 2018;360:254‐258. 29674575 10.1126/science.360.6386.254

[12] Zeier M , Reiser J . suPAR and chronic kidney disease‐a podocyte story. Pflugers Arch. 2017;469:1017‐1020. 28689240 10.1007/s00424-017-2026-7

[13] Wei C , El Hindi S , Li J , et al. Circulating urokinase receptor as a cause of focal segmental glomerulosclerosis. Nat Med. 2011;17:952‐960. 21804539 10.1038/nm.2411 PMC4089394

[14] Babelova A , Jansen F , Sander K , et al. Activation of Rac‐1 and RhoA contributes to podocyte injury in chronic kidney disease. PLoS ONE. 2013;8:e80328. 24244677 10.1371/journal.pone.0080328 PMC3820652

[15] Carriero MV , Bifulco K , Minopoli M , et al. UPARANT: a urokinase receptor‐derived peptide inhibitor of VEGF‐driven angiogenesis with enhanced stability and in vitro and in vivo potency. Mol Cancer Ther. 2014;13:1092‐1104. 24705350 10.1158/1535-7163.MCT-13-0949

[16] Dal Monte M , Rezzola S , Cammalleri M , et al. Antiangiogenic effectiveness of the urokinase receptor‐derived peptide UPARANT in a model of oxygen‐induced retinopathy. Invest Ophthalmol Vis Sci. 2015;56:2392‐2407. 25766585 10.1167/iovs.14-16323

[17] Cammalleri M , Dal Monte M , Locri F , et al. The urokinase receptor‐derived peptide UPARANT mitigates angiogenesis in a mouse model of laser‐induced choroidal neovascularization. Invest Ophthalmol Vis Sci. 2016;57:2600‐2611. 27168367 10.1167/iovs.15-18758

[18] Motta C , Lupo G , Rusciano D , et al. Molecular mechanisms mediating antiangiogenic action of the urokinase receptor‐derived peptide UPARANT in human retinal endothelial cells. Invest Ophthalmol Vis Sci. 2016;57:5723‐5735. 27787560 10.1167/iovs.16-19909

[19] Boccella S , Panza E , Lista L , et al. Preclinical evaluation of the urokinase receptor‐derived peptide UPARANT as an anti‐inflammatory drug. Inflamm Res. 2017;66:701‐709. 28456844 10.1007/s00011-017-1051-5

[20] Rezzola S , Corsini M , Chiodelli P , et al. Inflammation and N‐formyl peptide receptors mediate the angiogenic activity of human vitreous humour in proliferative diabetic retinopathy. Diabetologia. 2017;60:719‐728. 28083635 10.1007/s00125-016-4204-0

[21] Cammalleri M , Locri F , Marsili S , et al. The urokinase receptor‐derived peptide UPARANT recovers dysfunctional electroretinogram and blood‐retinal barrier leakage in a rat model of diabetes. Invest Ophthalmol Vis Sci. 2017;58:3138‐3148. 28632880 10.1167/iovs.17-21593

[22] Cammalleri M , Dal Monte M , Locri F , et al. Diabetic retinopathy in the spontaneously diabetic Torii rat: pathogenetic mechanisms and preventive efficacy of inhibiting the urokinase‐type plasminogen activator receptor system. J Diabetes Res. 2017;2017:2904150. 29464181 10.1155/2017/2904150 PMC5804371

[23] Tamma G , Procino G , Strafino A , et al. Hypotonicity induces aquaporin‐2 internalization and cytosol‐to‐membrane translocation of ICln in renal cells. Endocrinology. 2007;148:1118‐1130. 17138647 10.1210/en.2006-1277

[24] U.S. Department of Health and Human Services, Food and Drug Administration, Center for Drug Evaluation and Research . Guidance for industry, bioanalytical method validation. Silver Spring, MD: U.S. Department of Health and Human Services, Food and Drug Administration, Center for Drug Evaluation and Research; 2001 https://www.fda.gov/downloads/Drugs/Guidance/ucm070107.pdf.

[25] Scott RP , Quaggin SE . Review series: the cell biology of renal filtration. J Cell Biol. 2015;209:199‐210. 25918223 10.1083/jcb.201410017 PMC4411276

[26] Shi GJ , Shi GR , Zhou JY , et al. Involvement of growth factors in diabetes mellitus and its complications: a general review. Biomed Pharmacother. 2018;101:510‐527. 29505922 10.1016/j.biopha.2018.02.105

[27] Abd El Motteleb DM , Abd El Aleem DI . Renoprotective effect of *Hypericum perforatum* against diabetic nephropathy in rats: insights in the underlying mechanisms. Clin Exp Pharmacol Physiol. 2017;44:509‐521. 28079268 10.1111/1440-1681.12729

[28] Shukla R , Pandey N , Banerjee S , Tripathi YB . Effect of extract of *Pueraria tuberosa* on expression of hypoxia inducible factor‐1α and vascular endothelial growth factor in kidney of diabetic rats. Biomed Pharmacother. 2017;93:276‐285. 28648975 10.1016/j.biopha.2017.06.045

[29] Hou Y , Li S , Wu M , et al. Mitochondria‐targeted peptide SS‐31 attenuates renal injury via an antioxidant effect in diabetic nephropathy. Am J Physiol Renal Physiol. 2016;310:F547‐F559. 26719366 10.1152/ajprenal.00574.2014

[30] Maezawa Y , Takemoto M , Yokote K . Cell biology of diabetic nephropathy: roles of endothelial cells, tubulointerstitial cells and podocytes. J Diabetes Investig. 2015;6:3‐15. 10.1111/jdi.12255 PMC4296695 25621126

[31] Li P , Lu Q , Jiang W , et al. Pharmacokinetics and pharmacodynamics of rhubarb anthraquinones extract in normal and disease rats. Biomed Pharmacother. 2017;91:425‐435. 28475921 10.1016/j.biopha.2017.04.109

[32] Dong D , Sun H , Wu Z , et al. A validated ultra‐performance liquid chromatography‐tandem mass spectrometry method to identify the pharmacokinetics of SR8278 in normal and streptozotocin‐induced diabetic rats. J Chromatogr B Analyt Technol Biomed Life Sci. 2016;1020:142‐147. 10.1016/j.jchromb.2016.03.033 27038650

[33] Eddy AA , Fogo AB . Plasminogen activator inhibitor‐1 in chronic kidney disease: evidence and mechanisms of action. J Am Soc Nephrol. 2006;17:2999‐3012. 17035608 10.1681/ASN.2006050503

[34] De Blasio MJ , Ramalingam A , Cao AH , et al. The superoxide dismutase mimetic tempol blunts diabetes‐induced upregulation of NADPH oxidase and endoplasmic reticulum stress in a rat model of diabetic nephropathy. Eur J Pharmacol. 2017;807:12‐20. 28438648 10.1016/j.ejphar.2017.04.026

[35] Neymeyer H , Labes R , Reverte V , et al. Activation of annexin A1 signalling in renal fibroblasts exerts antifibrotic effects. Acta Physiol (Oxf). 2015;215:144‐158. 26332853 10.1111/apha.12586

[36] Wei C , Möller CC , Altintas MM , et al. Modification of kidney barrier function by the urokinase receptor. Nat Med. 2008;14:55‐63. 18084301 10.1038/nm1696

[37] Zhang B , Xie S , Shi W , Yang Y . Amiloride off‐target effect inhibits podocyte urokinase receptor expression and reduces proteinuria. Nephrol Dial Transplant. 2012;27:1746‐1755. 22076430 10.1093/ndt/gfr612

[38] Persson F , Theilade S , Eugen‐Olsen J , et al. Renin angiotensin system blockade reduces urinary levels of soluble urokinase plasminogen activator receptor (suPAR) in patients with type 2 diabetes. J Diabetes Complications. 2016;30:1440‐1442. 27475262 10.1016/j.jdiacomp.2016.07.003

[39] Bifulco K , Longanesi‐Cattani I , Liguori E , et al. A urokinase receptor‐derived peptide inhibiting VEGF‐dependent directional migration and vascular sprouting. Mol Cancer Ther. 2013;12:1981‐1993. 23939376 10.1158/1535-7163.MCT-13-0077

[40] Dimas GG , Didangelos TP , Grekas DM . Matrix gelatinases in atherosclerosis and diabetic nephropathy: progress and challenges. Curr Vasc Pharmacol. 2017;15:557‐565. 28155628 10.2174/1570161115666170202162345

[41] Jie L , Pengcheng Q , Qiaoyan H , et al. Dencichine ameliorates kidney injury in induced type II diabetic nephropathy via the TGF‐β/Smad signalling pathway. Eur J Pharmacol. 2017;812:196‐205. 28633927 10.1016/j.ejphar.2017.06.024

[42] Kolset SO , Reinholt FP , Jenssen T . Diabetic nephropathy and extracellular matrix. J Histochem Cytochem. 2012;60:976‐986. 23103723 10.1369/0022155412465073 PMC3527883

[43] Kenichi M , Masanobu M , Takehiko K , et al. Renal synthesis of urokinase type‐plasminogen activator, its receptor, and plasminogen activator inhibitor‐1 in diabetic nephropathy in rats: modulation by angiotensin‐converting‐enzyme inhibitor. J Lab Clin Med. 2004;144:69‐77. 15322501 10.1016/j.lab.2004.04.002

[44] Lin Y , Peng N , Zhuang H , et al. Heat shock proteins HSP70 and MRJ cooperatively regulate cell adhesion and migration through urokinase receptor. BMC Cancer. 2014;14:639. 25175595 10.1186/1471-2407-14-639 PMC4159539

[45] Parrish AR . Matrix metalloproteinases in kidney disease: role in pathogenesis and potential as a therapeutic target. Prog Mol Biol Transl Sci. 2017;148:31‐65. 28662825 10.1016/bs.pmbts.2017.03.001

[46] Kamiya Y , Iwai S , Nara K , et al. Effects of green tea on matrix metalloproteinases in streptozotocin‐induced diabetic rats. J Clin Biochem Nutr. 2005;37:77‐85.

[47] Geng J , Yu X , Liu C , et al. Herba artemisiae capillaris extract prevents the development of streptozotocin‐induced diabetic nephropathy of rat. Evid Based Complement Alternat Med. 2018;2018:5180165. 29636780 10.1155/2018/5180165 PMC5832121

[48] Zou C , Liu X , Liu R , et al. Effect of the oral iron chelator deferiprone in diabetic nephropathy rats. J Diabetes. 2017;9:332‐340. 27121697 10.1111/1753-0407.12420

[49] Zhang L , Li R , Shi W , et al. NFAT2 inhibitor ameliorates diabetic nephropathy and podocyte injury in db/db mice. Br J Pharmacol. 2013;170:426‐439. 23826864 10.1111/bph.12292 PMC3834765

[50] Lin Y , Zhang T , Feng P , et al. Aliskiren increases aquaporin‐2 expression and attenuates lithium‐induced nephrogenic diabetes insipidus. Am J Physiol Renal Physiol. 2017;313:F914‐F925. 28228402 10.1152/ajprenal.00553.2016 PMC6148297

[51] Kim Y , Park CW . New therapeutic agents in diabetic nephropathy. Korean J Intern Med. 2017;32:11‐25. 28049280 10.3904/kjim.2016.174 PMC5214729

[52] Tong F , Liu S , Yan B , et al. Quercetin nanoparticle complex attenuated diabetic nephropathy via regulating the expression level of ICAM‐1 on endothelium. Int J Nanomedicine. 2017;12:7799‐7813. 29123394 10.2147/IJN.S146978 PMC5661459

[53] Ahad A , Mujeeb M , Ahsan H , Siddiqui WA . Prophylactic effect of baicalein against renal dysfunction in type 2 diabetic rats. Biochimie. 2014;106:101‐110. 25151412 10.1016/j.biochi.2014.08.006

[54] Lawrence T . The nuclear factor NF‐kappaB pathway in inflammation. Cold Spring Harb Perspect Biol. 2009;1:a001651. 20457564 10.1101/cshperspect.a001651 PMC2882124

[55] Das R , Philip S , Mahabeleshwar GH , et al. Osteopontin: it's role in regulation of cell motility and nuclear factor kappa B‐mediated urokinase type plasminogen activator expression. IUBMB Life. 2005;57:441‐447. 16012053 10.1080/15216540500159424

[56] Dai Y , Palade P , Wang X , et al. High fat diet causes renal fibrosis in LDLr‐null mice through MAPK‐NF‐κB pathway mediated by Ox‐LDL. J Cardiovasc Pharmacol. 2014;63:158‐166. 24220312 10.1097/FJC.0000000000000035

[57] Galkina E , Ley K . Leukocyte recruitment and vascular injury in diabetic nephropathy. J Am Soc Nephrol. 2006;17:368‐377. 16394109 10.1681/ASN.2005080859

[58] Xie X , Peng J , Huang K , et al. Polydatin ameliorates experimental diabetes‐induced fibronectin through inhibiting the activation of NF‐κB signaling pathway in rat glomerular mesangial cells. Mol Cell Endocrinol. 2012;362:183‐193. 22732364 10.1016/j.mce.2012.06.008

[59] Dellamea BS , Leitão CB , Friedman R , Canani LH . Nitric oxide system and diabetic nephropathy. Diabetol Metab Syndr. 2014;6:17. 24520999 10.1186/1758-5996-6-17 PMC3928920

[60] Lin Q , Geng Y , Lin S , Tian Z . Sirtuin1 (SIRT1) regulates tumor necrosis factor‐alpha (TNF‐α‐Induced) aquaporin‐2 (AQP2) expression in renal medullary collecting duct cells through inhibiting the NF‐κB pathway. Med Sci Monit Basic Res. 2016;22:165‐174. 27980322 10.12659/MSMBR.901909 PMC5189724

[61] Bohuslavova R , Cerychova R , Nepomucka K , Pavlinkova G . Renal injury is accelerated by global hypoxia‐inducible factor 1 alpha deficiency in a mouse model of STZ‐induced diabetes. BMC Endocr Disord. 2017;17:48. 28774305 10.1186/s12902-017-0200-8 PMC5543752

[62] Alpers CE , Hudkins KL . Pathology identifies glomerular treatment targets in diabetic nephropathy. Kidney Res Clin Pract. 2018;37:106‐111. 29971205 10.23876/j.krcp.2018.37.2.106 PMC6027807

