# Supplementary material for: Inhibiting the urokinase‐type plasminogen activator receptor system recovers STZ‐induced diabetic nephropathy
Source: J Cell Mol Med. 2018 Nov 13;23(2):1034–49. doi: 10.1111/jcmm.14004 (PMC6349167; doi:10.1111/jcmm.14004)
Supplement: Supplementary file 1 [file JCMM-23-1034-s001.docx]

**Table S1.** Primary antibodies used in the Western blot analysis

| **Antibody** | **Dilution** | **Source** | **Catalogue** |
| --- | --- | --- | --- |
| Rabbit monoclonal anti-uPA | 1:1000 | Abcam | ab 133563 |
| Rabbit polyclonal anti-uPAR | 1:500 | Abcam | ab 103791 |
| Goat polyclonal anti-FPR1 | 1:200 | Santa Cruz Biotechnology | sc-13198 |
| Rabbit polyclonal anti-FPR2 | 1:200 | Santa Cruz Biotechnology | sc-66901 |
| Rabbit polyclonal anti-FPR3 | 1:200 | Santa Cruz Biotechnology | sc-66899 |
| Rabbit monoclonal anti αvβ3 integrin | 1:1000 | Novus Biologicals | NBP2-67557 |
| Rabbit polyclonal anti pβ3 integrin | 1:500 | Abcam | ab 38460 |
| Mouse monoclonal anti Rac-1 | 1:1000 | Abcam | ab 33186 |
| Rabbit polyclonal anti-plasminogen | 1:500 | Abcam | ab 154560 |
| Rabbit polyclonal anti-plasmin | 1:500 | Abcam | ab 48350 |
| Rabbit polyclonal anti-MMP-2 | 1:500 | Abcam | ab 37150 |
| Rabbit monoclonal anti-MMP-9 | 1:5000 | Abcam | ab 76003 |
| Rabbit polyclonal anti-fibronectin | 1:1000 | Abcam | ab 2413 |
| Rabbit polyclonal anti-collagen I | 1:000 | Abcam | ab 34710 |
| Rabbit polyclonal anti-collagen IV | 1:000 | Abcam | ab 6586 |
| Goat polyclonal anti-ZO1 | 1:200 | Santa Cruz Biotechnology | sc-8146 |
| Rat monoclonal anti-occludin | 1:200 | Millipore | 550274 |
| Rabbit polyclonal anti-VEGF | 1:200 | Santa Cruz Biotechnology | sc-507 |
| Mouse monoclonal anti-GFAP | 1:1000 | Sigma-Aldrich | G3893 |
| Rabbit polyclonal anti-AQP2 | 1:200 | Santa Cruz Biotechnology | sc-28629 |
| Rabbit polyclonal anti-iNOS | 1:200 | Santa Cruz Biotechnology | sc-8310 |
| Goat polyclonal anti-ICAM-1 | 1:200 | Santa Cruz Biotechnology | sc-1511 |
| Rabbit polyclonal anti-pNF-kB p65 (Ser^276^) | 1:200 | Santa Cruz Biotechnology | sc-101749 |
| Rabbit polyclonal anti-NF-kB p65 | 1:200 | Santa Cruz Biotechnology | sc-372 |
| Goat polyclonal anti-pCREB (Ser^133^) | 1:200 | Santa Cruz Biotechnology | sc-7978 |
| Rabbit polyclonal anti-CREB | 1:200 | Santa Cruz Biotechnology | sc-25785 |
| Rabbit polyclonal anti-HIF-1α | 1:200 | Santa Cruz Biotechnology | sc-10790 |
| Mouse monoclonal anti-β-actin | 1:25000 | Sigma-Aldrich | A2228 |
