# Supplementary material for: Inhibiting the urokinase‐type plasminogen activator receptor system recovers STZ‐induced diabetic nephropathy
Source: J Cell Mol Med. 2018 Nov 13;23(2):1034–49. doi: 10.1111/jcmm.14004 (PMC6349167; doi:10.1111/jcmm.14004)
Supplement: Supplementary file 2 [file JCMM-23-1034-s002.docx]

**Table S2.** Pharmacokinetic parameters of UPARANT in control and STZ rats.

|  | **control** | **STZ** |
| --- | --- | --- |
| **C_0_ (mg/L)** | 11 | 10 |
| **C_max_ (mg/L)** | 2.6 | 2.3 |
| **t_max_ (h)** | 2.3 | 2.2 |
| **k_abs_ (h^-1^)** | 0.6 ± 0.2 | 0.6 ± 0.2 |
| **k_elim_ (h^-1^)** | 0.31 ± 0.05 | 0.32 ± 0.05 |
| **AUC_inf_ (mg h/L)** | 15.9 | 16.1 |
| **t_1/2_ (h)** | 2.2 | 2.2 |

A two phase model equation C_t_ = C_0_(e^-kelim t^-e^-kabs t^) with first order kinetics (absorption and elimination) was used to evaluate the kinetic parameters. Both C_0_ corresponding to the plasma concentration extrapolated at t=0 from the elimination phase and k_elim_ and k_abs_, elimination and absorption kinetic parameters, were determined by best fit to the equation of the averaged time point concentrations. The terminal half-life t_1/2_ corresponding to 0.693/k_elim_ and time t_max_ at the maximum plasma concentration C_max_ were determined from the first derivative equal zero of the kinetic equation. The AUC_inf_, area under the concentration curve *versus* time from 0 to infinity was calculated by the integral of the kinetic equation.
